# Mobile phone app Vs bucket test as a subjective visual vertical test: a validation study

**DOI:** 10.1186/s40463-020-0402-3

**Published:** 2020-02-05

**Authors:** Tianyang Dai, George Kurien, Vincent YW Lin

**Affiliations:** 1 Faculty of Medicine, University of Toronto, 1 King’s College Cir, Toronto, ON M5S 1A8 Canada; 2 Department of Otolaryngology-Head and Neck Surgery, Sunnybrook Health Sciences Centre, 2075 Bayview Ave, Toronto, Ontario M4N 3M5 Canada

**Keywords:** Mobile app, Subjective visual vertical test, Validation study, Bucket test

## Abstract

**Background:**

The SVV tests the ability of a person to perceive the gravitational vertical. A tilt in SVV indicates vestibular imbalance in the roll plane, and thus injuries to the utricle or its connecting nerves. A validated bedside method (et, al., 2009, 72(19):1689–1692, Neurol, Zwergal) is the bucket method, in which the subject estimates the true vertical by attempting to properly align a straight line visible on the bottom of a bucket that is rotated at random by the examiner. In our study, the subjects need to align the plumb line on the Visual Vertical iOS app to the vertical direction.

**Methods:**

Measurements of the SVV were made in 22 healthy subjects (16 females and 6 males). Each subject conducted 10 iterations of bucket test and 10 iterations of iOS app test. The reliability and validity of the iOS app was analyzed by SPSS21.

**Results:**

Cronbach’s α for the plumb line method was 0.976, and the iOS app was 0.978. Statistical comparison of SVV values measured by the iOS app and the bucket method showed no significant difference in distribution (Mann Whitney U test U = 0.944).

**Conclusion:**

The Visual Vertical iOS app is an effective and accessible substitute to the plumb line for the measurement of the validated bucket test.

## Background

In conjunction with a complete and thorough history and physical exam, various vestibular tests are often helpful in the diagnosis of peripheral vestibular conditions. Many of these tests such as computerized dynamic posturography have been cumbersome or difficult to access. However, technological advances have brought many tests into the clinical environment in an affordable and feasible manner. The Subjective Visual Vertical (SVV) is one such test, which tests the ability of a person to perceive the gravitational vertical. A tilt in SVV is the most prominent indication of vestibular imbalance in the roll plane, caused by injuries to the utricle or its connecting nerves [1]. There are many tests measuring SVV, including the hemispheric dome method [1], the light bar in the dark method [2], ocular VEMP (oVEMP) [3], a newly developed software system [4], a monocular portable device [5], and the bucket test [6]. Among them, the bucket test, [7, 8], is the simplest and most cost-effective to perform, and thought to have comparable results [6] to other more expensive methods in discriminating asymmetric utricular function.

The original description of the test uses a bucket with a plumb line on the outside for the examiner to determine the degree of tilt. This method, however, has its limitations due to potential errors in measurement from parallax. A newly developed mobile phone application (Visual Vertical, iOS, Clearhealth Media, Wonga Park, Australia) has the potential to make this test more readily usable as well as accurate. This study aims to validate the iOS (Apple, Cupertino, USA) app in comparison to the previously validated bucket test.

## Methods

**Fig. 1:**
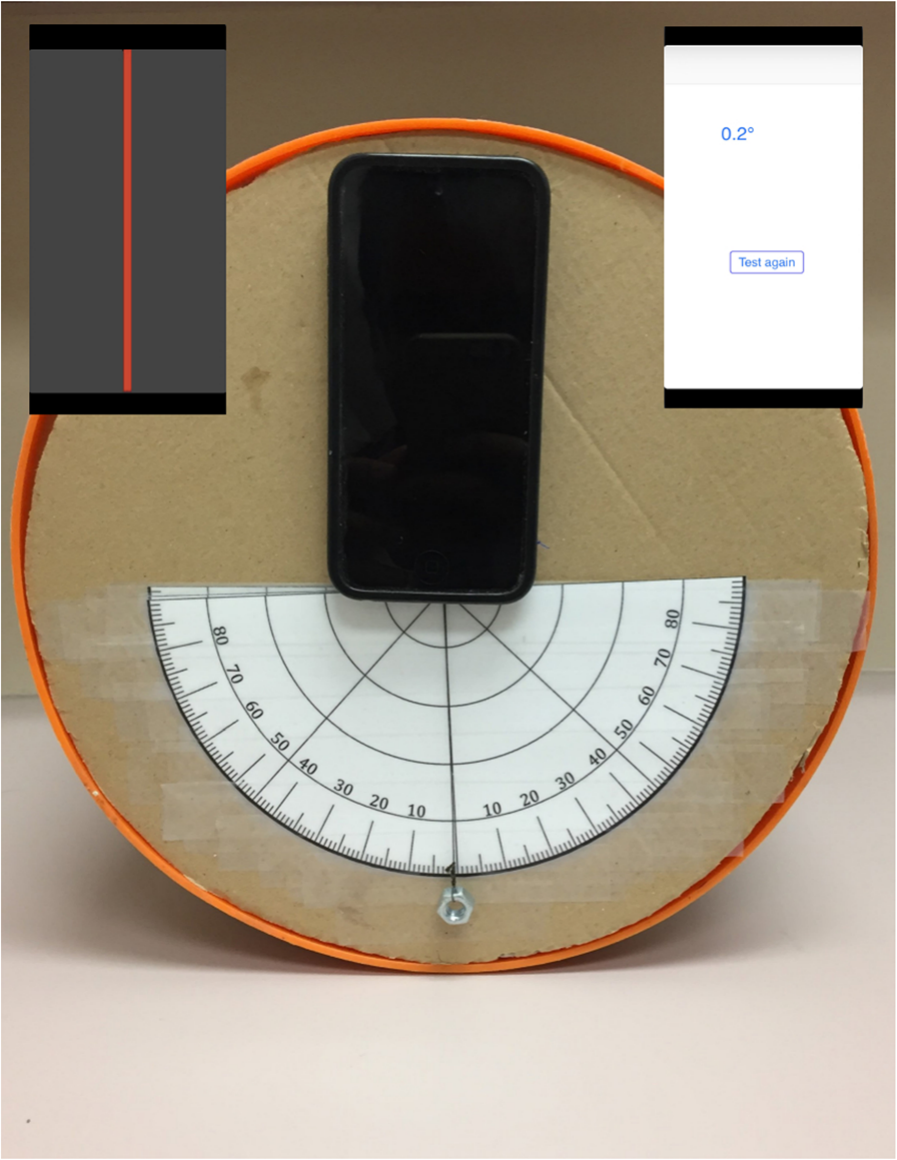
On the exterior base of the bucket, a plumb line originating from the center projected over an 180^0^ protractor, which is aligned with the straight diametric line on the inside base of the bucket. The iPod Touch with Visual Vertical was secured such that it was aligned with the zero- line of the degree scale

A total of twenty-two healthy volunteers (16 females and 6 males) were included in the study, all of whom underwent 10 iterations of the SVV test with the validated bucket method [6]. No volunteers had any history of otologic or vestibular conditions or pathology. A straight diametric line was prepared on the interior base of a bucket. On the exterior base of the bucket, a plumb line originating from the center projected over a 180^0^ protractor was aligned with the straight diametric line on the interior base of the bucket. Construction was as based on the work of Zwergal [6]. Additionally, on the exterior base of the bucket, an iPod Touch with Visual Vertical was secured such that it was aligned with the zero-line of the degree scale (Fig. 1). The iPod Touch was secured on the exterior base of the bucket with hook and loop fastener tape above the plumb line to compare the two measurement methods (plumb line and app). In a clinical setting, the iOS device can be secured on the interior base of the bucket alone and the subject would look at the vertical line on the device.

The examiner first calibrated the iOS app to the true vertical (determined as the plumb line) as the position of the iOS device varied slightly when being taken on and off for charging. This was done by recording the difference in degrees between the two methods when the plumb line was set to zero based on the examiner’s inspection. This value was then used to correct the final reading on the iOS device. For each iteration of the test, the bucket was then rotated to the left or right (in no predictable order). The subject was instructed to look into the bucket with their visual field completely covered by the rim of the bucket, return the diametric line to gravitational vertical by estimation. The subject was allowed 10 s by a timer on the app, which then produced a result. Both the manual reading on the scale and the measurement on the iOS app were simultaneously recorded by the examiner. Statistical analysis was performed using SPSS 21 (IBM Corp. Armonk, NY) to assess the reliability and validity of the iOS app.

## Results

The mean ± SD result of the plumb line method was 0.330 ± 1.37°, and the mean ± SD result of the iOS app was 0.350 ± 1.34°. Statistical comparison of SVV values measured by the iOS app method and the bucket method showed no significant difference in distribution (Mann Whitney U test U = 0.944). Cronbach’s alpha for the plumb line method was 0.976, and the iOS app was 0.978. The standard deviation of the 10 iterations from each subject was also calculated. The mean of all such standard deviations for the plumb line method is 0.654, compared to 0.620 for the iOS app method. The standard deviations of iOS app method are statistically smaller than those of the plumb line method (t = − 2.187, df = 21, *p* = 0.04).

## Discussion

The Visual Vertical iOS app is an effective and accessible substitute to the plumb line for the measurement of subjective visual vertical. The statistically insignificant mean difference and high result correlation between the plumb line and the iOS app validates that the app produces comparable results to the plumb line. The app, however, reports the results of tests with a time limit in each trial (option of 10, 15, or 20 s). In contrast, the traditional plumb line method allows the subjects to spontaneous control the amount of time they would like to report the results when they are ready. With a similarly high Cronbach’s alpha, the internal reliability of the app is arguable superior to the plumb line method. In addition, the standard deviations of the 10 iterations from each subject in the app method are statistically smaller than those of the plumb line (0.620 Vs 0.654, *p* = 0.04), indicating that the app is a test of higher precision. The app also provides a reading precision of 0.1, in contrast to the traditional degree scale used in plumb line method with a precision of 0.5. Furthermore, the app assisted bucket test is more accessible given the prevalence of compatible devices, easier to read (no parallax error) and maximizes the inter-rater reliability, particularly in examiners with visual acuity problems. One disadvantage of the app is its dependence of accuracy on the internal gyroscope of the iOS device. Regular maintenance of the device is required to avoid the circumstances of dis-calibration, which is more costly and inconvenient than the plumb line method. The limitation of the study is that it was performed in a healthy subject population with no prior vestibular pathology or complaints. However, given that the test procedure was the same as that of the already validated bucket test with plumb line method, the results obtained are applicable in validating the new (iOS app) method. The next step in validation would be that of testing in patients with pathologic processes affecting utricular function.

## Conclusion

Overall, the Visual Vertical iOS app demonstrates a similar level of precision and accuracy as the validated plumb line method, with few compromises in its clinical utility. Although further assessments need to be conducted, it offers a convenient and efficient alternative to conduct the bucket test.

## Data Availability

The datasets used and/or analyzed during the current study are available from the corresponding author on reasonable request.

## Abbreviations

SVV: Subjective visual vertical test
VEMP: Vestibular evoked myogenic potential
